# Pyrosequencing, a method approved to detect the two major EGFR mutations for anti EGFR therapy in NSCLC

**DOI:** 10.1186/1756-9966-30-57

**Published:** 2011-05-16

**Authors:** Sandrine Dufort, Marie-Jeanne Richard, Sylvie Lantuejoul, Florence de Fraipont

**Affiliations:** 1 UM Biochimie des Cancers et Biothérapies, CHU Grenoble, Institut de Biologie et Pathologie, parvis Belledonne, 38 043 Grenoble, France; 2 Centre de recherche INSERM/UJF U823, Institut Albert Bonniot, Rond-point de la Chantourne, 38 709 La Tronche cedex 9, France; 3 Département d'Anatomie et Cytologie Pathologiques, CHU Grenoble, Institut de Biologie et Pathologie, parvis Belledonne, 38 043 Grenoble, France

## Abstract

**Background:**

Epidermal Growth Factor Receptor (EGFR) mutations, especially in-frame deletions in exon 19 (ΔLRE) and a point mutation in exon 21 (L858R) predict gefitinib sensitivity in patients with non-small cell lung cancer. Several methods are currently described for their detection but the gold standard for tissue samples remains direct DNA sequencing, which requires samples containing at least 50% of tumor cells.

**Methods:**

We designed a pyrosequencing assay based on nested PCR for the characterization of theses mutations on formalin-fixed and paraffin-embedded tumor tissue.

**Results:**

This method is highly specific and permits precise characterization of all the exon 19 deletions. Its sensitivity is higher than that of "BigDye terminator" sequencing and enabled detection of 3 additional mutations in the 58 NSCLC tested. The concordance between the two methods was very good (97.4%). In the prospective analysis of 213 samples, 7 (3.3%) samples were not analyzed and EGFR mutations were detected in 18 (8.7%) patients. However, we observed a deficit of mutation detection when the samples were very poor in tumor cells.

**Conclusions:**

pyrosequencing is then a highly accurate method for detecting ΔLRE and L858R EGFR mutations in patients with NSCLC when the samples contain at least 20% of tumor cells.

## Introduction

Detection of mutations of the epidermal growth factor receptor (EGFR) gene is critical for predicting the response to therapy with tyrosine kinase inhibitors (TKIs, e.g.: gefitinib and erlotinib) in patients with non-small-cell lung cancer (NSCLC) [1]. Practically all mutations are on exons 18 through 21 where they affect the ATP-binding cleft of EGFR [2]. *In vitro *studies have shown that EGFR mutants have constitutive TK activity and, therefore, a greater sensitivity to anti-EGFR inhibition. Two classes of mutation account for approximately 90% of EGFR mutations reported to date in lung adenocarcinoma [3]. The class I mutations are in-frame deletions in exon 19, which almost always include amino-acid residues leucine 747 to glutamic acid 749 (ΔLRE). The second mutation is a single-point mutation in exon 21, which substitutes an arginine for a leucine at codon 858 (L858R).

Thus far, the direct DNA sequencing method is the most common and conventional method used for the detection and identification of mutations in tumor cells. However, its sensitivity is suboptimal for clinical tumor samples. Mutant DNA needs to comprise ≥25% of the total DNA to be easily detected [4]. All new techniques claim to be more sensitive with the ability to detect mutations in samples containing ≤10% mutant alleles. Pyrosequencing is a non-electrophoretic real time sequencing technology with luminometric detection [5]. Not only can it detect mutations but it also permits a mutation to be characterized and to quantify the percentage of mutated alleles in a sample. We have previously shown that it is a robust method to characterize the KRAS codon 12 and 13 mutations in paraffin-embedded samples in daily practice [6].

Here we also show that pyrosequencing is a simple and sensitive method to detect the two most common mutations of the EGFR TK domain, and demonstrate its usefulness for detecting such mutations in clinical lung tumor samples, in a large prospective series.

## Materials and methods

### Cell lines

The human lung cancer cell lines NCI-H1650 and NCI-H1975 were obtained from the American Type Culture Collection (ATCC). Both cell lines were cultured in RPMI 1640 supplemented with 10% fetal bovine serum at 37°C in air containing 5% CO_2_. Peripheral Blood Lymphocytes (PBL) used as negative control were obtained from healthy volunteers.

### Clinical samples

Between 1^st ^January and 30 June 2010, 213 tumor samples were collected from consecutive patients with an advanced lung adenocarcinoma, DNA extracted and their EGFR mutation status determined for selection for anti EGFR treatments by clinicians. All analyses were conducted with full respect of patients' rights to confidentiality and according to procedures approved by the local authorities responsible for ethics in research. All samples were histologically analyzed by an experienced thoracic pathologist and classified according to the WHO classification of lung cancer. For each sample, the percent of tumor cells was determined.

### DNA extraction

The DNAeasy kit (Qiagen) was used according to the manufacturer's instructions to extract genomic DNA from cells and from tumor tissues. A prolonged (48H) proteinase K digestion was used for paraffin-embedded tissues [6].

### PCR amplification of exons 19 and 21 of the EGFR gene

PCR and sequencing primers were designed using the PSQ assay design (Biotage) and are described in table 1. 100 ng of tumor DNA was amplified using a nested PCR to amplify almost all samples independent of the type of tissue fixative or of the fixative conditions. The first PCR product was amplified at 58°C for 20 (exon 19) or 10 (exon 21) cycles. The second PCR procedure was carried out in a total volume of 50 μl containing 2 μl of the first PCR, 20 pmol of each primer, 1.5 mmol/l MgCl_2 _and 1.25 U of FastStart Taq DNA polymerase (Roche). PCR conditions consisted of initial denaturing at 95°C for 15 min, 45 cycles at 95°C for 20 s, 62°C (exon 19) or 61°C (exon 21) for 20 s, 72°C for 20 s and a final extension at 72°C for 10 min. The PCR products (10 μl) were analyzed by electrophoresis in a 3% agarose gel to confirm the successful amplification of the 180-bp or the 195-bp PCR product.

**Table 1:** Sequences of primers used for pyrosequencing analysis

	Exon 19		Exon 21	
	primer sequence	T° of hybridation	primer sequence	T° of hybridation
First	5'-GCAATATCAGCCTTAGGTGCGGCTC-3'	58°C	5'-CTAACGTTCGCCAGCCATAAGTCC-3'	58°C
PCR	5'-CATAGAAAGTGAACATTTAGGATGTG-3'		5'-GCTGCGAGCTCACCCAGAATGTCTGG-3'	

second	5'-CATGTGGCACCATCTCACAAT-3'	62°C	5'-GAATTCGGATGCAGAGCTTCTT-3'	61°C
PCR	5'-Biotin-CCCACA CAGCAA AGCAGAAACT-3'		5'-Biotin-CTTTCTCTTCCGCACCCA	

primer for sequence reaction	5'-TAAAATTCCCGTCGC-3'		5'-CATGTCAAGACTACAGATT-3'	

### Pyrosequencing analysis

40 μl of PCR product were bound to streptavidin Sepharose HP (GE Healthcare), purified, washed, denatured using a 0.2 mol/l NaOH solution, and washed again. Then 0.3 μmol/l pyrosequencing primer was annealed to the purified single-stranded PCR product and the pyrosequencing was performed on a PyroMark ID system (Qiagen) following the manufacturer's instructions. The nucleotide dispensation order was GTATCAGACATGAC for analysis of exon 19 and CTGCGTGTCA for analysis of exon 21.

## Results

### Pyrosequencing assay of exon 19 deletions

**Figure 1:**
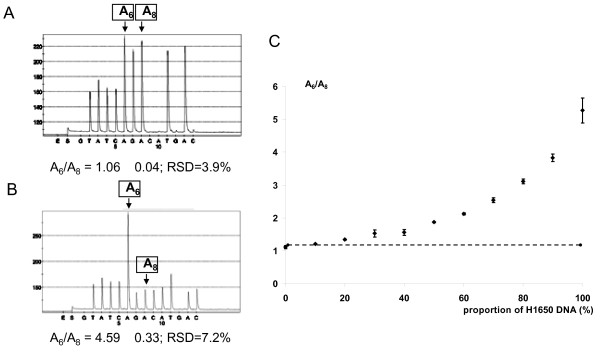
**Analysis of exon 19 deletions by pyrosequencing**. The analysis was performed with PBL DNA (A) as wild-type control and with NCI-H1650 DNA (B) as deletion control. The deletion was quantified by determining the ratio between the A_8 _and A_6 _peak areas. (C) The sensitivity was characterized by measuring A8/A6 ratio in different mixtures of NCI-H1650 DNA and PBL DNA.

**Figure 2:**
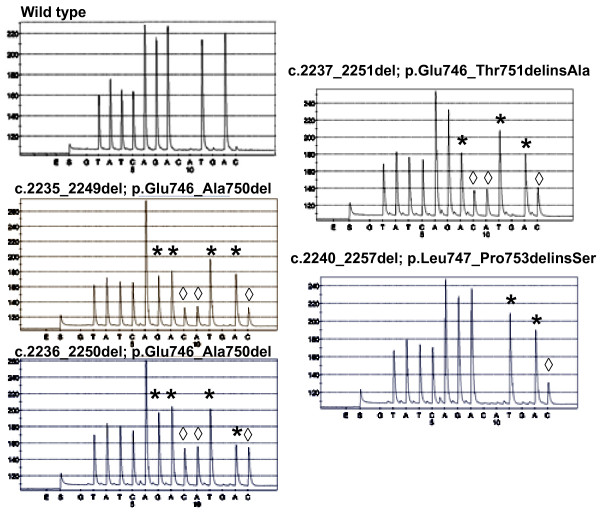
**Comparison of different pyrograms observed for exon 19 analyses in different tumor tissues**. The exon 19 status were described as wild type or deleted (*: peak diminished in the deleted samples; ◊: peak increased in the deleted samples).

In order to test the pyrosequencing method for the analysis of exon 19 deletions, we used DNA from the NCI-H1650 cell line as positive control and DNA extracted from human peripheral blood lymphocytes (PBL) as wild-type control. We choose a particular pyrosequencing program with the oligonucleotide dispensation order (GTATCAGACATGAC) because it permits to distinguish wild type and mutated alleles (table 2) generating for each sample a specific pyrogram (Figure 1A and 1B and Figure 2). These pyrograms correspond to a mix of wild type and mutated alleles. We quantitatively evaluated the exon 19 deletion (c.2235-2249del; p.Glu746-Ala750del) by determining the ratio between the peak areas of the two adenines dispensed in positions 6 (A_6_) and 8 (A_8_). We tested the reproducibility of the technique by analyzing each DNA in 20 consecutive and independent runs. We found an A_6_/A_8 _ratio of 1.06 ± 0.04 for the wild type sample and 4.59 ± 0.33 for the sample with the deletion. The relative standard deviation (RSD) was respectively 3.9% and 7.2%. Thus, a sample could be considered as mutated if A_6_/A_8 _was superior to 1.2 (corresponding to [the mean + 3 standard deviations] of the wild type sample). To demonstrate the assay sensitivity, we also quantified the A_6_/A_8 _ratio in various mixtures (10/0, 9/1, 8/2, 7/3, 6/4, 5/5, 4/6, 3/7, 2/8, 1/9 and 0/10) of DNA from the NCI-H1650 cell line with DNA from peripheral blood lymphocytes (Figure 1C). Each mixture was analyzed 5 times in the same run and we found an A_6_/A_8 _ratio varying from 5.27 ± 0.38 (mixture 10/0) to 1.11 ± 0.05 (mixture 0/10). We determined that all the mixtures containing at least 20% of NCI-H1650 DNA have an A_6_/A_8 _ratio superior to 1.2 and could be considered as mutated.

**Table 2:** Sequencing of wild type and mutated alleles with a particular program of pyrosquencing

nucleotide dispensation during pyrosequencing		G	T	A	T	C	A	G	A	C	A	T	G	A	C
	WT		**T**	**A**	**T**	**C**	**AA**	**GG**	**AA**			**TT**		**AA**	
allelic	c.2235-2249del		**T**	**A**	**T**	**C**	**AA***AA*			*C*	*A*	*T*			*C*
sequence of	c.2236-2250del		**T**	**A**	**T**	**C**	**AA**	**G**	*A*	*C*	*A*	*T*			*C*
	c.2237-2251del		**T**	**A**	**T**	**C**	**AA**	**GG**		*C*	*A*	*T*			*C*
	c.2240-2257del		**T**	**A**	**T**	**C**	**AA**	**GG**	**AA**			**T**			*C*

Bold letters correspond to the nucleotides identical in wild type and mutated alleles; italic letters correspond to the nucleotides specific of mutated alleles.

Moreover, the pyrosequencing program that analyzed the deletions in exon 19 was designed to detect almost all types of deletion (figure 2). In comparison with the graph obtained with the wild type sample, the diminution of several peaks (marked *) and the emergence of new ones (marked ◊) were considered as specific of a deletion (table 2).

### Pyrosequencing assay of L858R exon 21 point mutation

**Figure 3:**
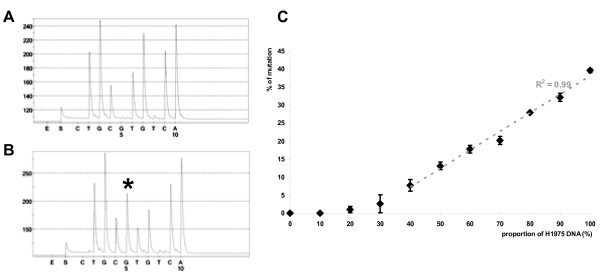
**Analysis of c.2573T > G; p.Leu858Arg exon 21 mutation by pyrosequencing**. Examples of pyrosequencing profiles obtained with PBL (A) and NCI-H1975 (B) DNA. * represented the T > G mutation. (C) Sensitivity curve established with different mixtures of NCI-H1975 and PBL DNA.

L858R-specific pyrosequencing was performed using the NCI-H1975 cell line and a percentage of T > G mutation was determined (Figure 3). The result obtained with 20 consecutive runs, was 46.2 ± 3% with good reproducibility (RSD = 6.4%). We also determined the repeatability and the sensitivity of this method with various mixtures (10/0, 9/1, 8/2, 7/3, 6/4, 5/5, 4/6, 3/7, 2/8, 1/9 and 0/10) of DNA from the NCI-H1975 cell line and DNA from peripheral blood lymphocytes (Figure 3C). We detected the percentage of T > G mutation with a linear variation (R^2 ^= 0.99) from 39.6 ± 0.6% (mixture 10/0) to 7.7 ± 1.7% (mixture 4/6) and a relative standard deviation varying from 1.4 to 15.9%. We also determined a% of mutation for the mixtures 3/7 and 2/8 with a CV largely higher then 20%.

### EGFR mutation in tumor samples

We compared the results obtained previously by conventional BigDye Terminator sequencing [7] using the method described by Pao et al [8] and those obtained by pyrosequencing 58 of these tumor samples (Table 3). All mutated samples were confirmed twice, starting from independent polymerase chain reactions. We observed a very high concordance between the two methods (56/58 (96.6%) for exon 19 and 57/58 (98.3%) for exon 21 analysis). For 3 samples (3/58; 5%), results were discordant and mutations were detected only by pyrosequencing and not by Big Dye terminator sequencing, reflecting the lower sensitivity of the classical sequencing method. Indeed, the two samples with an exon 19 deletion have an A_6_/A_8 _ratio of 1.7 and 1.8 which correspond to less of 25% of mutated alleles (figure 1C). For the sample with a L858R mutation detected only by pyrosequencing, we found that only 22.5% of the DNA was mutated.

**Table 3:** Comparison of EGFR status (wild type (WT) or mutant (M)) of exon 19 and exon 21 determined by big dye sequencing or by pyrosequencing on 58 NSCLC tissues

Exon 19		big dye sequencing		Exon 21		big dye sequencing	
		WT	M			WT	M
pyrosequencing	WT	47	/	pyrosequencing	WT	53	/
	M	2	9		M	1	4

We then determined the EGFR status of 213 patients with advanced or metastatic lung adenocarcinomas for selection of to anti EGFR therapies (table 4). Seven (3.3%) samples were inconclusive due to poor DNA quality with no DNA amplification. Of the 206 remaining samples, 18 EGFR mutations were detected (8 of exon 19 and 10 of exon 21) (18/206; 8.7%). Among these 206 specimens, 36 had less than 20% of tumor cells and only one with a mutation was detected (1/36; 2.8%). For the 170 specimens containing more than 20% of tumor cells, 17 with mutations were found (17/170; 10%).

**Table 4:** Prospective evaluation of the EGFR status of exons 19 and 21

% of tumoral	tumoral samples (n = 206)		EGFR mutations (n = 18)		
cells	number	%	exon 19	exon 21	%
<20%	36	17.5	0	1	2.8
from 20 to 50%	98	47.6	3	6	9.2
>50%	72	35	5	3	11.1

Samples may contain at least 20% of tumor cells to allow a correct detection of mutations

## Discussion

Pyrosequencing is sensitive and enables accurate detection of mutations. A previous study has described the capacity of this method to detect small insertions [9] but this study is the first to demonstrate the application of pyrosequencing to exon 19 deletions. Analysis of exon 21 by pyrosequencing had been succinctly described by Takano et al. [10,11], but without any data about the specificity, the repeatability or the sensitivity.

We first investigated the characteristics of EGFR mutations in the lung cancer cell lines NCI-H1650 and NCI-H1975 and used them as positive controls for the deletion in exon19 and the point mutation in exon 21 respectively. Moreover we used the DNA of these cells mixed with DNA isolated from blood samples from healthy volunteers to evaluate the basic properties of our novel method. We didn't observe strict linearity because the two cell lines (NCI-H1650 and NCI-H1975) have respectively 4 and 2.8 EGFR gene copies [12] but we found good sensitivity.

In routine daily practice fixed paraffin-embedded specimens, most often of small size, are the only samples available for both diagnosis and molecular analyses. The DNA is frequently fragmented, which could hamper PCR amplification. However, the PCR conditions described in this study allowed analysis of 96.7% of the paraffin-embedded tissues whatever the type of fixative used or the duration of the fixation. When the samples could be amplified and analyzed, results were concordant (97.4%) with those obtained by conventional BigDye terminator sequencing. The difference in sensitivity between the two methods is illustrated by the 3 samples characterized as mutated only by pyrosequencing. The frequency of deletions in exon 19 and mutations in exon 21 among the NSCLC patients was almost consistent with the corresponding values reported in Caucasian populations [13]. These samples were also analyzed for KRAS mutations because (i) EGFR and KRAS mutations are mutually exclusive in NSCLC and (ii) emerging data suggest that KRAS mutations are negative predictors of benefit from both adjuvant chemotherapy and anti-EGFR-directed therapies [12,14,15]. We found 26.7% of the samples with a KRAS mutation (data not shown). This is also in accordance with the literature [14] and validated our cohort as being well representative. We found 8 exon 19 deletions and 10 exon 21 mutations. These results were in accordance with those described by Tanaka et al. [16]. They noticed that exon 19 deletions were significantly associated with a male gender. In our cohort, 15 of the 18 patients with EGFR mutations were female.

We observed a deficit in mutation detection when the samples were very poor in tumor cells whereas the others could be accurately analyzed. As only bronchial or trans-thoracic fine needle biopsies are usually available in the medical setting of patients with advanced stage NSCLC (around 90% of the samples analyzed here, with only 10% being surgical specimens), these results demonstrate the need for a pathologist's expertise to qualify the samples and perform microdissection if samples contain less than 20% of tumor cells. Indeed, Masago et al. [17] have demonstrated that results could be obtained from biopsy specimens only if the quantity of the specimen is sufficient to make a pathological diagnosis and if cancer cells were carefully selected. However, microdissection is very time-consuming and it is not always possible. Alternatively, methods such as peptide nucleic acid-locked nucleic acid PCR clamp [18,19] or real-time PCR based on scorpion primers coupled with the Amplified Refractory Mutation System (ARMS) [20] have a sensitivity around 1% of cancer cells. However, they could be difficult to use in routine clinical assay because they require special equipments and expensive reagents.

## Conclusions

The present pyrosequencing method is sufficiently sensitive and specific to enable the detection of the two major TKI-sensitive mutations in a large majority of the DNA extracted from paraffin-embedded clinical samples.

## Competing interests

The authors declare that they have no competing interests.

## Authors' contributions

SD carried out the molecular analysis, MJR participated in the design of the study and drafted the manuscript, SL carried out immunohistochemestry analysis, FdeF designed the study, carried out the molecular analysis and drafted the manuscript. All authors reviewed the draft manuscript, read and approved the final version for submission.
